# Extracellular vesicle-associated Aβ mediates trans-neuronal bioenergetic and Ca^2+^-handling deficits in Alzheimer’s disease models

**DOI:** 10.1038/npjamd.2016.19

**Published:** 2016-09-22

**Authors:** Erez Eitan, Emmette R Hutchison, Krisztina Marosi, James Comotto, Maja Mustapic, Saket M Nigam, Caitlin Suire, Chinmoyee Maharana, Gregory A Jicha, Dong Liu, Vasiliki Machairaki, Kenneth W Witwer, Dimitrios Kapogiannis, Mark P Mattson

**Affiliations:** 1 Laboratory of Neurosciences, National Institute on Aging, NIH, Baltimore, MD, USA; 2 Sanders-Brown Center on Aging, and Department of Neurology, University of Kentucky College of Medicine, Lexington, KY, USA; 3 Department of Pathology, Johns Hopkins University School of Medicine, Baltimore, MD, USA; 4 Department of Molecular and Comparative Pathobiology and Department of Neurology, Johns Hopkins University School of Medicine, Baltimore, MD, USA; 5 Department of Neuroscience, Johns Hopkins University School of Medicine, Baltimore, MD, USA

## Abstract

Alzheimer’s disease (AD) is an age-related neurodegenerative disorder in which aggregation-prone neurotoxic amyloid β-peptide (Aβ) accumulates in the brain. Extracellular vesicles (EVs), including exosomes, are small 50–150 nm membrane vesicles that have recently been implicated in the prion-like spread of self-aggregating proteins. Here we report that EVs isolated from AD patient cerebrospinal fluid and plasma, from the plasma of two AD mouse models, and from the medium of neural cells expressing familial AD presenilin 1 mutations, destabilize neuronal Ca^2+^ homeostasis, impair mitochondrial function, and sensitize neurons to excitotoxicity. EVs contain a relatively low amount of Aβ but have an increased Aβ42/ Aβ40 ratio; the majority of Aβ is located on the surface of the EVs. Impairment of lysosome function results in increased generation of EVs with elevated Aβ42 levels. EVs may mediate transcellular spread of pathogenic Aβ species that impair neuronal Ca^2+^ handling and mitochondrial function, and may thereby render neurons vulnerable to excitotoxicity.

## Introduction

Alzheimer’s disease (AD) is a progressive neurodegenerative disease that accounts for 60–70% of cases of dementia in the aging population. The disease has two pathological hallmarks: extracellular amyloid β (Aβ) peptide aggregates that form extracellular plaques, and intracellular hyperphosphorylated Tau protein aggregates that form neurofibrillary tangles. Pathogenic forms of Aβ and Tau can impair synaptic function and trigger a series of events leading to neuronal death.^1^ Although the causes of Aβ and Tau aggregation and cytotoxicity in the common sporadic late-onset cases of AD are unclear, 1–5% of AD patients exhibit early-onset disease resulting from mutations in the amyloid precursor protein (APP) or presenilin 1 (PS1; an enzyme that cleaves APP to generate Aβ).^1,2^ Studies of the consequences of expressing AD PS1 and APP mutations in cultured cells and transgenic mice have provided insight into the molecular and cellular alterations underlying the pathogenesis of AD.^3^ In particular, aggregating Aβ and Tau can impair mitochondrial function and perturb neuronal Ca^2+^ handling, thereby rendering synapses and neurons vulnerable to degeneration.^4–8^

Recently, it has been suggested that pathogenic forms of Aβ and Tau can propagate between cells in a prion-like manner, such that a misfolded protein can pass from a donor to a recipient cell and serve as a seed for further protein conversion and aggregation.^9,10^Although it has been shown that injection of Aβ from either biological or synthetic sources into the mouse brain can accelerate endogenous Aβ aggregation throughout the brain, the mechanism responsible for this propagation of the pathology is unclear.^11^ Extracellular vesicles (EVs), including exosomes, are small-membrane vesicles released from all cell types and found in bodily fluids, are one potential mechanism by which misfolded proteins and protein aggregates might spread through tissues.^12,13^ EVs can contain several aggregation-prone proteins responsible for neurodegenerative disease including Aβ,^14^ APP C-terminal fragments,^15^ Tau,^16^ α-synuclein,^17^ SOD1^18^ and the prion protein.^19^ Moreover, two EV markers, Alix and Flotillin, have been found in amyloid plaques, suggesting a role of EVs in plaque generation.^14,20^ EVs are secreted by either direct budding of the plasma membrane or fusion of multivesicular bodies with the plasma membrane.^21^ Multivesicular bodies have been reported as an important site of Aβ generation, leading to the suggestion that EVs may also be a site of APP processing into Aβ.^14,22^ Interestingly, EVs released by healthy neurons can interact with extracellular Aβ and facilitate its fibrillation and clearance by microglia.^23–26^

Although EVs from AD patients and various AD models contain some amount of Aβ (refs 14,15,27) the contribution of EVs to Aβ production and the pathogenesis of AD is not clear. Here we report that EVs isolated from the cerebrospinal fluid (CSF) of patients with sporadic, late-onset AD or released from cultured human neurons harboring a pathogenic PS1 mutation are toxic to cerebral cortical neurons by a mechanism involving EV surface-associated Aβ. EVs with neurotoxic Aβ impair neuronal Ca^2+^ handling and mitochondrial function. The release of EVs with neurotoxic Aβ is increased when lysosome function is compromised, suggesting that the impaired lysosome function that occurs in neurons in AD^28^ may trigger EV-mediated trans-neuronal propagation of neurodegenerative cascades.

## Results

### Extracellular vesicles from PS1 mutant cells contain a relatively low concentration of Aβ, but are enriched in Aβ42 relative to Aβ40

**Figure 1:**
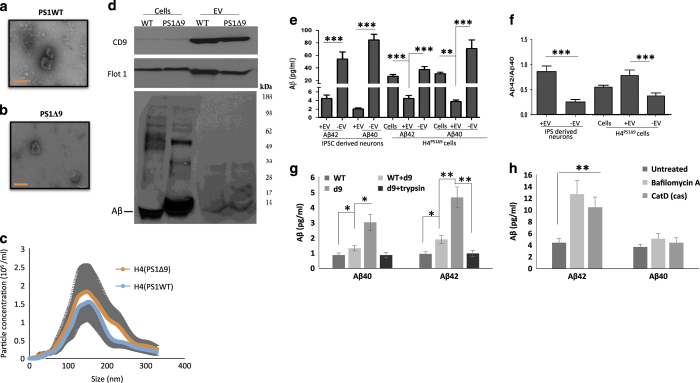
Human neural cells expressing familial Alzheimer’s disease (AD) presenilin 1 mutations release extracellular vesicles (EVs) with elevated levels of Aβ42 on their outer surface. (**a**, **b**). Transmission electron microscope images of EVs released from H4 cells expressing either the *δ*9 PS1 mutation (**a**) or wild-type (WT) PS1 (**b**) Bar = 100 nm. (**c**) Size distribution of EVs released from H4 cells expressing mutant (red) or wild type (blue) PS1 measured by Nanocyte particle tracking. (**d**) Immunoblot demonstrating enrichment of EV markers CD9 and Flotillin-1 in EVs released from H4 cells, and relatively low amounts of amyloid β-peptide (Aβ) in EVs relative to H4 cell lysate. (**e**, **f**) Levels of Aβ42 and Aβ40 (**e**) and the Aβ42/Aβ40 ratio (**f**) in cells, EVs and EV-depleted medium from neurons differentiated from induced pluripotent stem cell (iPSC) that were generated from fibroblasts from a patient with familial AD (PS1 mutation; *n*=5 cultures), and from H4 human neuroglioma cells expressing mutant PS1 (8 separate cultures). (**g**) Levels of Aβ40 and Aβ42 in EVs isolated from the medium of cultured H4Ps1Δ9 cells, H4Ps1WT cells, H4Ps1WT EVs incubated in H4Psn1Δ9 EV-depleted medium, or H4Psn1Δ9 EVs incubated with trypsin (*n*=3). (**h**) Levels of Aβ40 and Aβ42 in EVs isolated from the medium of cultured H4Psn1Δ9 cells that had been treated for 24 h with vehicle (control; *n*=5) or bafilomycin A (*n*=5), or in which cathepsin D was knocked down using CRISPR Cas9 technology (*n*=3). *P<0.05, **P<0.01, ***P<0.001.

To investigate the effects of pathogenic PS1 mutations on EV-associated Aβ, we first isolated EVs from the culture medium of H4 glioblastoma cell lines genetically engineered to express either the PS1Δ9 mutation (H4^PS1Δ9^) or wild-type PS1 (H4^PS1WT^) in an inducible manner. The expression of PS1Δ9 or PS1WT was induced by tetracycline for 5 days, and EVs were isolated from the culture medium by ultracentrifugation.^29^ The EVs were evaluated according to the published guidelines^30^ by transmission electron microscopy, immunoblot analysis and NTA particle size analysis (Figure 1a–d). The vast majority EVs were 50–250 nm in diameter, with the most abundant size being about 130 nm (Figure 1c). The EVs contained high amounts of the EV marker proteins flotillin-1 and CD9 (Figure 1d). We found that the PS1Δ9 mutation did not alter EV morphology, size distribution or expression of these EV markers. The levels of Aβ40 and Aβ42 associated with EVs and free in the culture medium were measured by a Mesoscale Discovery (MSD, Rockville, MD, USA) immunoassay (Figure 1e). Aβ40 and Aβ42 were significantly lower in the EV fraction than in the EV-depleted medium fraction and in the parent cell lysates (Figure 1e). Given that the Aβ42/Aβ40 ratio may be a better predictor of AD than the individual concentrations of Aβ40 and Aβ42, we calculated the Aβ42/Aβ40 ratio^31,32^ and found that the Aβ42/Aβ40 ratio in EVs was significantly higher than in EV-depleted medium or cell lysate (Figure 1f).

To further characterize the association of Aβ with EVs, we examined EVs released from cultured human neurons derived from induced pluripotent stem cells generated from fibroblasts taken from a patient with AD caused by a PS1 mutation (hN^PS1A246E^ neurons) and control neurons generated from fibroblasts from a neurologically normal human subject.^33^ EVs isolated from culture medium were evaluated by NTA and immunoblotting with antibodies against the EV markers flotillin-1 and CD9 (Supplementary Figure 1). The levels of Aβ42 and Aβ40 associated with EVs were significantly lower than Aβ42 and Aβ40 levels in the EV-depleted medium (Figure 1e), but the Aβ42/Aβ40 ratio was significantly higher in EVs compared to EV-depleted medium (Figure 1f). As expected, the levels of Aβ42 were higher in H4^PS1Δ9^ cell lysate, culture medium and EVs compared to H4^PS1WT^ cells, and the level of Aβ42 was higher in hN^PS1A246E^ medium than hN^PS1WT^ medium (Supplementary Figure 2).

We next determined whether EV-associated Aβ is contained within EVs and/or on the outer surface of their membrane. EVs were incubated with 1 mg/ml trypsin for 1 h prior to two rounds of 120,000*g* centrifugation to purify EVs. The trypsin treatment reduced the amount of Aβ42 and Aβ40 associated with EVs isolated from H4^PS1Δ9^ cells by 82% (Figure 1g). These results indicate that most EV-associated Aβ is located on the outer surface of the EV membrane. It has been reported previously that neuronal EVs contain several Aβ-binding proteins and lipids.^34–36^ Thus, it is possible that the Aβ is not released together with the EVs but becomes associated with EVs extracellularly. However, incubating EVs derived from H4^PS1WT^ cells with EV-depleted medium derived from H4^PS1Δ9^ resulted in only a very small increase in the levels of EV-associated Aβ (Figure 1g), suggesting that Aβ becomes associated with vesicles prior to their release from cells.

Accumulating evidence indicates that Aβ production can occur in the endosomal system, including within multivesicular bodies.^14,22,37^ We hypothesized that if the association of Aβ with vesicles occurs in the endosomal system, inhibiting the lysosome would increase the release of Aβ42-containing EVs.^38^ Incubating H4^PS1Δ9^ cells for 24 h with 200 μmol/l of bafilomycin A significantly increased EV secretion by 1.4-fold (data not shown), and the concentration of Aβ associated with EVs was increased by about 3-fold (Figure 1h); the Aβ42/Aβ40 ratio was also increased significantly (Supplementary Figure 3). Bafilomycin A is a V-ATPase inhibitor and, while being a potent inhibitor of lysosomal function, it also has an effect on endosomal pH levels, which could affect the activity of β-secretase and γ-secretase.^38,39^ Therefore, we measured the effect of bafilomycin A on Aβ concentration in the cells and culture medium. The level of Aβ in the cells was increased similarly to that in EVs, but there was no significant difference in the level of Aβ in culture media from control and bafilomycin A-treated cells (Supplementary Figure 3A). To verify the effect of lysosome inhibition on EV-associated Aβ, we employed Cas9 with a sgRNA targeted to disrupt the cathepsin D gene (Supplementary Figure 3B). H4 cells, in which cathepsin D was knocked down, secreted EVs with threefold more Aβ and had a higher Aβ42/Aβ40 ratio compared with EVs secreted from control H4 cells (Figure 1h and Supplementary Figure 3C).

### EVs derived from PS1 mutant cells are neurotoxic

**Figure 2:**
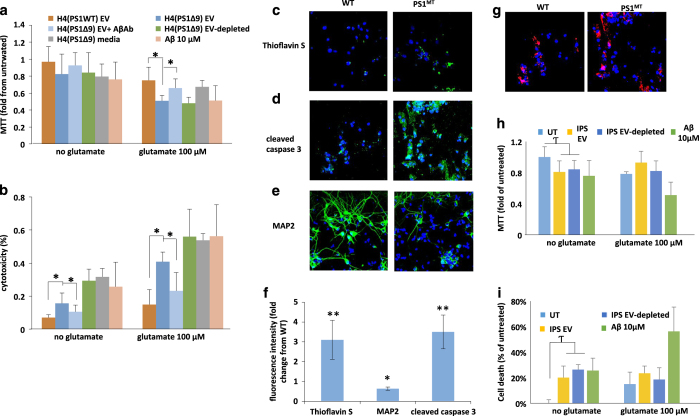
Extracellular vesicles (EVs) isolated from the medium of cultured human neural cells expressing mutant PS1 are neurotoxic. (**a**, **b**) Rat primary cortical neurons were exposed to the indicated treatments for 24 h and cell viability was evaluated by MTT (**a**) and LDH (**b**) assays (*n*=3–6 separate cultures). (**c**) Thioflavin S (green), and 4′,6-diamidino-2-phenylindole dihydrochloride (DAPI; blue) staining demonstrates that treating cortical neuron cultures with Psn1Δ9-derived EVs results in the accumulation of Thioflavin S reactive aggregates. (**d**) Cleaved caspase 3 staining (green) and DAPI (blue) staining showing increased cell death following incubation of cortical neuron cultures with Psn1Δ9-derived but not WT EVs. (**e**). Reduced MAP2 staining (green; a neuronal protein) is observed in rat neuronal cultures incubated with Psn1Δ9-derived EVs. DAPI (blue). (**f**). Quantification of fluorescence intensities of primary neuronal cultures that had been exposed for 48 h to EVs derived from H4 cells expressing mutant PS1 and then stained with Thioflavin S, cleaved caspase 3 and MAP2. Values are expressed as fold difference from the value for neurons exposed to EVs from control H4 cells (*n*=6 cultures). (**g**) Images showing PKH-labeled EVs (red) and DAPI (blue) staining in cortical neurons that had been incubated for 6 h in the presence of EVs released from H4 cells expressing WT PS1 or mutant PS1 (PS1MT). (**h**, **i**). Levels of MTT reduction and LDH release (cytotoxicity) in cortical neurons that that had been incubated for 48 h in the presence of EVs from neurons derived from induced pluripotent stem cell (iPSC) generated from fibroblasts from a patient with familial Alzheimer’s disease (AD; IPS), EV-depleted iPSC culture medium, or Aβ1–42 (10 μmol/l). UT, untreated control cultures. Cultures were co-treated with glutamate (100 nmol/l) or vehicle as indicated. Values are the mean and s.e.m. (*n*=6 cultures). *P<0.05, **P<0.01. Aβ, amyloid β-peptide.

Having established that PS1 mutant neurons release EVs that have higher levels of Aβ on their surface, we performed a series of experiments to determine whether these EVs are neurotoxic. When EVs isolated from the culture medium of H4^PS1Δ9^ cells were added to primary rat cortical neurons at a concentration of ~300 EVs/neuron, significant reductions of neuronal viability occurred within 48 h (Figure 2a,b). Interestingly, incubation of primary cortical neurons with EV-depleted medium (containing about 10-fold more Aβ42 than EVs) or with 10 μmol/l of recombinant Aβ42 had a similar effect on neuronal viability as did the lower amount of Aβ associated with EVs released from cells expressing mutant PS1 (Figure 2a,b). It was previously reported that Aβ42 sensitizes neurons to glutamate-induced excitotoxicity.^40^ We, therefore, determined whether EVs isolated from the medium of mutant PS1-expressing cells affect the vulnerability of neurons to excitotoxicity. Cortical neurons were exposed to EVs for 24 h, followed by the addition of 100 μmol/l glutamate for an additional 24 h. Parallel neuronal cultures were exposed to EVs, glutamate or vehicle for 48 h. Glutamate alone induced a 15–20% reduction in viability, but in combination with EVs from H4^PS1Δ9^ cells, neuronal viability was reduced by 50–56% (Figure 2a,b). To further examine whether the neurotoxicity of H4^PS1Δ9^ derived EVs is mediated by Aβ, the ability of an antibody against Aβ (6E10) to protect neurons was examined; the Aβ antibody abolished the neurotoxicity of the EVs (Figure 2a,b). The latter result suggests that the neurotoxic property of EVs is mediated by Aβ on their surface.

To further investigate the neurotoxicity of EV-associated Aβ, we incubated neurons for 48 h with EVs derived from H4^PS1Δ9^ cells followed by fixation and staining with Thioflavin S (to label Aβ aggregates with a β-sheet structure), and antibodies against cleaved-caspase 3 (a marker of apoptosis) and the neuron-specific cytoskeletal protein MAP2 (Figure 2c–f). The fluorescent intensities of both Thioflavin S and cleaved-caspase 3 were significantly greater (4- and 3.5-fold higher, respectively) in neurons treated with EVs from H4^PS1Δ9^ cells compared with neurons treated with EVs from H4^PS1wt^ cells, while the amount of MAP2 immunoreactivity was 40% lower (Figure 2f). The levels of Thioflavin S were also greater in neurons treated with H4^PS1wt^ derived EVs compared with untreated neurons. To determine the relative numbers of EVs internalized by the neurons, EVs were stained with the lipophilic dye PKH26 before incubation with cortical neurons, and internalized EVs were imaged by confocal microscopy (Figure 2g). There was no apparent difference in the amount of H4^PS1Δ9^ and H4^PS1WT^ EVs internalized by the neurons (Figure 2g and quantification in Supplementary Figure 4A). To better quantify the levels of internalization, different amounts of labeled EVs were added to the culture medium, and the increase in flourecence intensity was measured after 4 and 24 h of incubation. EV concentration-dependent increases in internalization was seen at both time points (Supplementary Figure 4B,C). Incubating the labeled EVs with Aβ antibody only slightly reduced their internalization (Supplementary Figure 4B,C) and thus the ability of the antibody to completely block neurotoxicity is probably not due to reduced internalization. EVs from hN^PS1A246E^ neurons were relatively less neurotoxic than EVs from H4^PS1Δ9^cells (Figure 2h,i). This difference could be due to the two different PS1 mutations (PS1 A246E compared with PS1Δ9), to different amounts or conformations of Aβ42 on the EV surfaces, or to differential biological activities of the EVs independent of Aβ (for example, it has been reported that EVs released from stem cells have trophic effects^41^).

### AD cell-derived exosomes cause calcium dysregulation and mitochondrial impairment, and can trigger neuronal apoptosis

**Figure 3:**
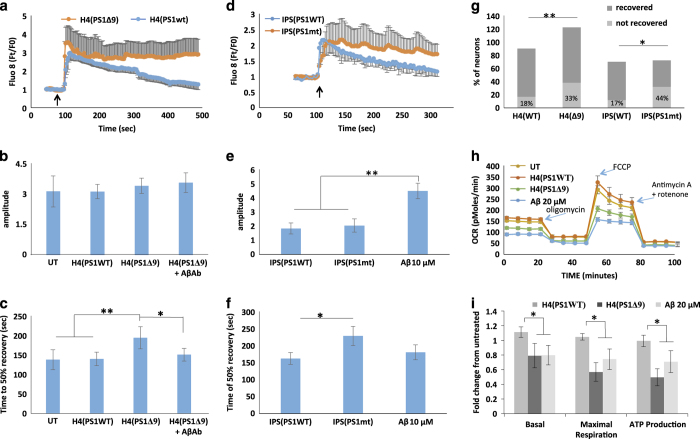
Extracellular vesicles (EVs) generated by human neural cells expressing mutant PS1 impair cellular Ca^2+^ handling and mitochondrial function in cerebral cortical neurons. (**a**) Preincubation of rat cortical neurons with H4Psn1Δ9-derived EVs for 24 h resulted in a greater sustained Ca^2+^ response to glutamate (10 μmol/l) stimulation (arrow indicates the time of glutamate application) compared with neurons preincubated with EVs from H4Psn1WT cells. Values are the mean and s.e.m. of measurements made in 20 neurons per culture in 7 different cultures. (**b**, **c**) Quantification of peak amplitude (**b**) and time to reach 50% recovery (**c**) following glutamate stimulation in cortical neurons that had been pretreated for 24 h with EVs (H4D9 cells with either wild type (WT) or Δ9 PS1; H4(PS1Δ9)+AβAb was a condition in which the EVs were incubated with an amyloid β-peptide (Aβ) antibody (6E10). Values are the mean and s.e.m. of measurements made in neurons from 7 different cultures. (**d**) Alzheimer’s disease (AD) patient iPSC-derived neuronal EVs induce Ca^2+^ dysregulation following stimulation with glutamate (10 μmol/l; arrow indicates the time of glutamate application). Values are the mean and s.e.m. of measurements made in three different cultures (20 neurons per culture). (**e**, **f**) Peak amplitude (**e**), and time to reach 50% recovery (**f**) following glutamate stimulation in rat cortical neurons pretreated for 24 h with EVs from AD patient or WT induced pluripotent stem cells (iPSC)-derived neurons (Aβ condition is rat cortical neurons incubated with 10 μmol/l synthetic Aβ1–42). (**g**). Analysis of the percentage of cortical neurons unresponsive to glutamate following incubation with mutant or wild-type (WT) EVs derived from H4 cells and IPSC-derived neurons (*n*=7 for H4 EVs treated neurons and *n*=3 for IPC EVs treated neurons). (**h**) Representative Seahorse oxygen consumption assay showing mitochondrial impairment 24 h following treatment with H4Psn1Δ9-derived EVs or 20 μmol/l Aβ1–42. (**i**) Basal oxygen-consumption rate, maximal consumption rate and ATP production in cortical neurons that had been treated for 24 h with H4Psn1Δ9-derived EVs or 20 μmol/l Aβ1–42. Values are the mean and s.e.m. from measurements made in five independent cultures. *P<0.05, **P<0.01.

To determine the cause of the observed neurotoxicity of EVs released from cells expressing AD mutant PS1, we examined the effects of these EVs on neuronal Ca^2+^ handling following glutamate exposure and on cellular metabolism using the Seahorse mitochondrial function assay (Agilent Technologies, Santa Clara, CA USA). Primary cortical neurons were incubated for 48 h with EVs released from H4^PS1Δ9^ or H4^PS1wt^ cells, or with EVs released from hN^PS1A246E^ neurons. The [Ca^2+^]_I_ responses to glutamate in neurons treated with EVs from H4^PS1Δ9^, H4^PS1wt^, hN^PS1wt^ neurons, hN^PS1A246E^ neurons or synthetic Aβ42 are shown in Figure 3a–f. Incubating neurons with EVs from PS1 mutant cells did not induce any increase in the magnitude of the peak [Ca^2+^]_I_ response (Figure 3b,e), but did cause a significant increase in the response duration (37% and 21% increases for EVs from H4^PS1Δ9^ cells and iPS neurons, respectively; Figure 3c,f). While adding the Aβ blocking antibody did not have an effect on amplitude of [Ca^2+^]_I_ response (Figure 3b), it did enhance recovery of [Ca^2+^]_I_ following glutamate exposure (Figure 3a,c), suggesting that the effect of EVs on recovery of [Ca^2+^]_I_ is mediated by the Aβ on the surface of the EVs. This analysis may underestimate the increase in [Ca^2+^]_I_ response duration caused by EVs, because the [Ca^2+^]_I_ in rat cortical neurons treated with EVs released from H4^PS1Δ9^ and induced pluripotent stem cells-derived neurons did not recover to 50% of maximum (Figure 3g).

We next performed a Seahorse mitochondrial function assay on neurons treated with EVs released from H4^PS1Δ9^ and H4^PS1wt^ cells, and found significantly reduced mitochondrial functionality as a result of incubation with EVs released from cells expressing the PS1Δ9 mutation (Figure 3h,i). The effects of H4^PS1Δ9^ cell-derived EVs in reducing basal and maximal respiration, and ATP levels, were similar to the reductions in these mitochondrial variables in neurons exposed to synthetic Aβ42 (Figure 3h,i). Collectively, the data are consistent with a role of EV surface-associated Aβ42 in the neurotoxic effects of EVs.

### Extracellular vesicles in mouse models of AD and AD patient CSF exhibit a high Aβ42/40 ratio

**Figure 4:**
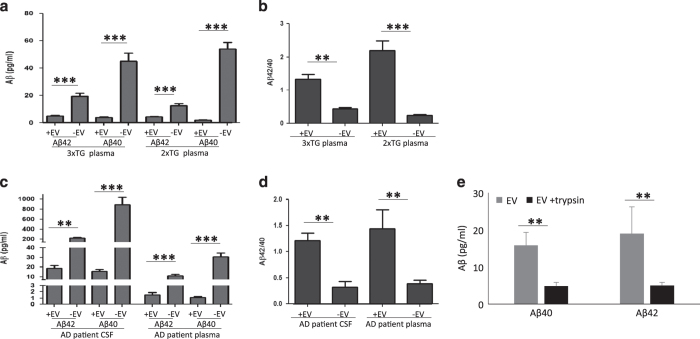
Extracellular vesicles (EVs) isolated from cerebropspinal fluid (CSF) and plasma of Alzheimer’s disease (AD) patients, and from plasma in two AD mouse models, exhibit a high Aβ42/40 ratio. Concentrations of Aβ40 and Aβ42 were measured in samples of EVs (+EV) and corresponding fluids from which they were isolated (−EV). (**a**, **b**). Results of analyses of blood plasma samples from 3xTgAD and 2xTgAD mice (*n*=5 3xTgAD and *n*=6 2xTgAD mice). (**c**, **d**). Results of analyses of CSF and blood plasma samples from AD patients (*n*=6 patients). (**e**) Quantification of Aβ40 and Aβ42 levels in EVs isolated from the CSF of AD patients. The EVs were incubated with or without with trypsin prior to the analysis (EV preparations from three different AD patients). **P<0.01, ***P<0.001. Aβ, amyloid β-peptide.

To determine whether EVs isolated from biological fluids of AD patients and animal models exhibited Aβ-related neurotoxic properties similar to EVs released from cultured cells expressing mutant PS1, we measured the concentrations of Aβ42 and Aβ40 in EVs isolated from plasma samples from six transgenic APP/PS1 double-mutant transgenic mice,^42^ five 3xTgAD mice^43^ and nine age-matched wild-type mice. The levels of Aβ42 and Aβ40 were significantly lower in EVs and EV-depleted plasma samples from wild-type mice compared with transgenic mice (Supplementary Figure 5). The levels of Aβ42 and Aβ40 were significantly lower in plasma-derived EVs than in EV-depleted plasma (Figure 4a), but the Aβ42/Aβ40 ratio was significantly higher in the EVs compared with EV-depleted plasma (Figure 4b). Next, we isolated EVs from CSF samples from six patients with sporadic, late-onset AD, six mild cognitive impairment (MCI) patients and six age-matched control subjects. EVs isolated from CSF were highly enriched in the EV markers flotillin-1 (FLOT1) and Alix (Supplementary Figure 6A), but not the intracellular (non-exosomal) protein early endosome antigen 1 (EEA1; data not shown). Interestingly, the EV size distribution in all CSF samples showed two populations, one with a mean diameter of about 70 nm, a size typical of exosomes, and another with a mean size of about 170 nm, which may be microvesicles.^21^ The concentration of EVs in the CSF of AD, MCI and healthy individuals were similar and much lower than their concentration in plasma (Supplementary Figure 6D).

We next measured the levels of Aβ42 and Aβ40 in CSF-derived EVs and found that these were significantly lower in EVs compared with EV-depleted CSF and plasma samples of AD patients (Figure 4c). The Aβ42/Aβ40 ratio was significantly higher in EVs derived from CSF and plasma of AD patients compared with EV-depleted CSF and plasma samples (Figure 4d). The levels of Aβ42 and Aβ40 associated with EVs were also low in CSF samples from subjects with MCI and aged-matched neurologically normal healthy subjects, but the Aβ42/Aβ40 ratio was significantly higher in EVs isolated from AD patients CSF compared with EVs from control subjects CSF (Supplementary Figure 7). To determine whether EV-associated Aβ from AD patient CSF was contained within EVs and/or on their surface, EVs were incubated with 1 mg/ml trypsin for 1 h prior to two rounds of 120,000*g* centrifugation to purify EVs. Similar to EVs released from H4^PS1Δ9^ cells (Figure 1g), we observed a 75% reduction in the amount of Aβ42 and Aβ40 associated with EVs treated with trypsin compared with EVs not treated with trypsin (Figure 4e).

### CSF-derived exosomes cause calcium dysregulation and mitochondrial impairment and can trigger neuronal apoptosis

**Figure 5:**
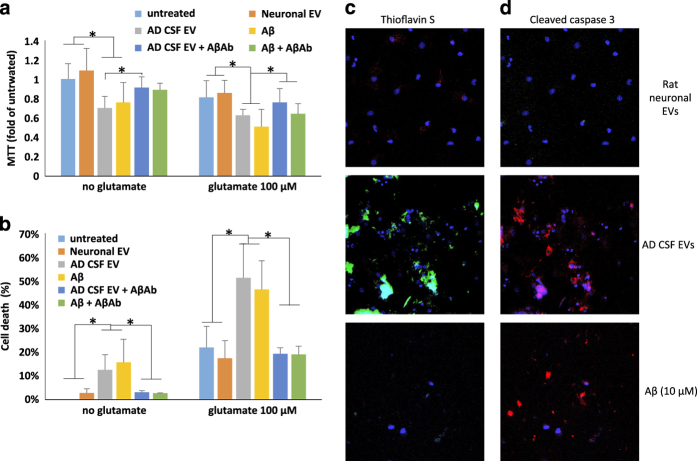
Alzheimer’s disease (AD) patient CSF-derived extracellular vesicles (EVs) are neurotoxic. (**a**, **b**) Results of MTT and LDH assays demonstrating that 48 h incubation of rat neurons with AD cerebrospinal fluid (CSF) EVs (at a concentration of 100 EVs per neuron) diminished neuronal survival by an amount similar to that of neurons exposed to Aβ1–42 (10 μmol/l). AD CSF EVs also increased neuronal vulnerability to glutamate during a 24 h incubation (*n*=6 cultures). Where indicated, cultures were co-treated with Aβ antibody 6E10 (1 μg antibody per 10^7^ EVs). (**c**, **d**) Cultured cortical neurons were incubated for 24 h in the presence of fluorescently tagged EVs that had been isolated from the culture medium of rat cortical neurons or from AD CSF (100 EVs per neuron), or with 10 μmol/l Aβ1–42. Cortical neurons were then stained with Thioflavin S or anti-cleaved caspase 3. Representative images are shown. Similar results were obtained in six separate experiments. *P<0.05. Aβ, amyloid β-peptide.

The finding of high Aβ42/Aβ40 ratio of CSF-derived EVs from patients with sporadic, late-onset AD prompted us to evaluate their potential neurotoxicity. EVs isolated from CSF were added to the medium bathing cultured cerebral cortical neurons (~100 EVs per neuron), and 48 h later cell viability was measured with MTT and LDH assays. Exposure of neurons to CSF-derived EVs resulted in a significant reduction of neuronal viability in MTT (Figure 5a) and LDH (Figure 5b) assays. The magnitude of reduction of viability of CSF EV-treated neurons was similar to that measured in neurons exposed to 10 μmol/l Aβ42, whereas EVs isolated from cultured rat cortical neurons exhibited no neurotoxicity (Figure 5a,b). Moreover, EVs isolated from the CSF of healthy human subjects did not induce significant toxicity (Supplementary Figure 7). CSF EVs from AD patients and synthetic Aβ42 significantly increased the vulnerability of cortical neurons to glutamate excitotoxicity, and these adverse effects of AD CSF EVs and synthetic Aβ42 were abolished by treatment with an Aβ antibody (Figure 5a,b). To determine whether EVs from AD CSF cause Aβ aggregation and apoptosis, neurons were stained with thioflavin S and a cleaved-caspase 3 antibody (Figure 5c,d). Thioflavin S staining was undetectable in untreated neurons (not shown) and in neurons treated for 48 h with EVs isolated from the medium of cultured rat cortical neurons, but was evident in neurons incubated for 48 h with human CSF-derived EVs, and to a lesser extent in neurons treated with Aβ1–42 (Figure 5c). Whereas no neurons exhibited cleaved-caspase 3 immunoreactivity in cortical neuron cultures treated with EVs released from healthy cortical neurons, many neurons exhibited cleaved caspase 3 immunoreactivity in cortical neurons treated with AD CSF-derived EVs or Aβ42 (Figure 5c,d).

To provide further insight into the role of EV-associated Aβ in neuronal degeneration in AD, we plotted the concentration of Aβ42 in EVs isolated from cell culture medium and human subject CSF as a function of the relative neurotoxicity of EVs from the same sources (Supplementary Figure 8). The most neurotoxic EVs, those from AD patient CSF and those released from cells expressing mutant PS1, also had the highest amounts of Aβ42 associated with them. In contrast, EVs released from control human cells were the least neurotoxic and had the lowest amounts of Aβ42 associated with them. There was a highly significant correlation of EV Aβ42 level and neurotoxicity of the EVs (Supplementary Figure 8).

**Figure 6:**
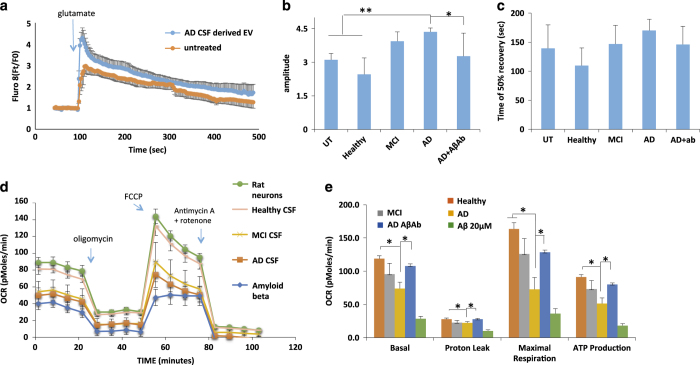
Alzheimer’s disease (AD) patient cerebrospinal fluid (CSF)-derived extracellular vesicles (EVs) impair neuronal Ca^2+^ regulation and mitochondrial function. (**a**) Graph showing relative intracellular Ca^2+^ levels (Fluo 8 fluorescence intensity before and during exposure to glutamate (100 μmol/l). (**b**, **c**) Graphs showing amplitudes of peak Ca^2+^ responses to glutamate (**b**) and the time required for the Fluo 8 fluorescence intensity to recover to 50% of the peak level (**c**). (**d**, **e**) Results of Seahorse analysis of mitochondrial respiration in cultured neurons that had been pretreated for 24 h with EVs isolated from the medium bathing healthy primary rat cortical neurons (100 particles per neuron), 10 μmol/l amyloid β-peptide (Aβ) or EVs isolated from the CSF of AD patients (100 particles per neuron). (**d**) Results of a representative experiment. (**e**) Data from six experiments. *P<0.05; **P<0.01.

We next performed Ca^2+^ imaging experiments in which [Ca^2+^]_I_ responses to glutamate were measured in vehicle-treated control rat cortical neurons and in neurons that had been preincubated for 48 h with CSF EVs from AD, MCI and control subjects, or Aβ42. Neurons incubated with AD CSF-derived EVs demonstrated a significant 25±9% increase in the peak amplitude of the glutamate-induced elevation of [Ca^2+^]_I_; this effect of AD CSF EVs was prevented by treatment with an Aβ antibody (Figure 6a,b). The time required for the [Ca^2+^]_I_ to return to 50% of the peak [Ca^2+^]_I_ response to glutamate was not significantly affected by AD CSF EVs, although there was a trend towards slower recovery of [Ca^2+^]_I_ (*P*<0.072; Figure 6c). We next measured neuronal oxygen consumption to evaluate mitochondrial function in cortical neurons exposed for 48 h to CSF EVs from AD, MCI and control subjects (Figure 6d shows the results of a representative experiment, and Figure 6e is the combined data from six experiments). Neurons treated with AD patient CSF-derived EVs or with Aβ42 had significantly reduced basal and maximal respiration, and reduced ATP production (Figure 6e). The adverse effects of AD CSF EVs on mitochondrial function were abolished by treatment with an Aβ antibody (Figure 6e).

## Discussion

We found that compared with various control EVs, EVs released from cultured human neurons and cell lines expressing mutant PS1, and EVs isolated from the CSF of sporadic, late-onset AD patients, have elevated amounts of Aβ42 (predominantly located on their surface) and can induce degeneration of cerebral cortical neurons. EVs from sporadic, late-onset AD patient CSF and EVs released from cells expressing mutant PS1 impaired mitochondrial function, destabilized neuronal Ca^2+^ homeostasis, and rendered cortical neurons vulnerable to excitotoxicity. The adverse effects of the AD patient and experimental model EVs on neuronal Ca^2+^ handling, mitochondrial function and cell viability were prevented by treatment with an Aβ antibody, indicating a key role for EV surface-associated Aβ in pathogenic effects of AD EVs.

Our findings provide novel insight into potential roles of EVs in propagating Aβ-related pathology and associated neuronal degeneration. It has been suggested that AD pathology spreads in a trans-neuronal manner with prion-like characteristics,^9,10^ and this possibility is supported by data from mouse model experiments where inoculation with synthetic Aβ or Aβ-containing brain homogenate accelerate development of pathology.^11^ Interestingly, we found that although the majority of Aβ in CSF, plasma and culture medium is not associated with EVs, the EVs have significantly higher Aβ42/Aβ40 ratio and are toxic to primary neurons, suggesting a possible role for EVs in AD pathogenesis. The toxic effect of EVs is specific to AD (even in sporadic, late-onset cases) or at least to neurodegenerative disease, because CSF EVs from healthy subjects exhibited little or no neurotoxicity.

We found that EVs isolated from CSF, plasma and culture medium from human neural cells expressing pathogenic PS1 mutations contained lower concentrations of Aβ40 and Aβ42 compared with the fluid from, which they were isolated. However, the Aβ42/Aβ40 ratio was significantly higher in EVs compared with the fluids from which they were isolated. These findings are of interest with regards to AD pathogenesis as the Aβ42/Aβ40 ratio has been found to be a more reliable biomarker for AD than Aβ42 or Aβ40 alone.^44^ Moreover, mixtures of Aβ42 and Aβ40 with a higher Aβ42/Aβ40 ratio aggregate more vigorously and are more neurotoxic than mixtures with a low Aβ42/Aβ40 ratio.^45,46^ It was recently reported that EVs of neuronal origin isolated from the blood of AD patients prior to their diagnosis contain elevated levels of Aβ42 (and p-Tau) compared with EVs from healthy control subjects.^47^ We found that EVs have a relatively high Aβ42/40 ratio and are potent in disrupting neuronal Ca^2+^ homeostasis, impairing mitochondrial function and triggering apoptosis. However, whether Aβ associated with EVs has a critical role in the pathogenesis of AD remains to be determined.

Recent findings suggest that EVs can transport pathogenic forms of Aβ, pTau and α-synuclein into recipient neurons via internalization.^14,16,17^ We found that EVs isolated from sporadic, late-onset AD patient CSF or from the culture medium of human cells expressing mutant PS1, are internalized by cerebral cortical neurons. The recipient neurons accumulate Thioflavin S-reactive protein aggregates, and manifest impaired Ca^2+^ handling and mitochondrial dysfunction. However, in contrast to the presumption that Aβ is located inside EVs,^13^ we found that Aβ1–42 is predominantly located on the external surface of EVs. Previous findings suggest that Aβ aggregation is enhanced when the pathogenic peptide is associated with cell membranes, particularly membranes rich in sphingomyelin, gangliosides and cholesterol, so-called lipid rafts.^48–52^ During the process of its aggregation on the membrane surface, Aβ can generate reactive oxygen species that induce membrane lipid peroxidation,^53,54^ which can impair the function of membrane ion-motive ATPases, and glucose and glutamate transporters, and thereby render neurons vulnerable to excitotoxicity and apoptosis.^55–57^ Our data suggest that similar to aggregating synthetic Aβ1–42, EV surface-associated Aβ can increase the vulnerability of neurons to excitotoxicity and apoptosis by a mechanism involving impaired Ca^2+^ regulation.

It has been suggested that neuronal EVs can scavenge extracellular Aβ by binding of Aβ to EV proteins such as cystatin c,^35^ insulin-degrading enzyme,^34,58^ the cellular prion protein^26^ and ganglioside GM1.^36^ We incubated EVs derived from cells expressing wild-type PS1 with EV-depleted medium from PS1Δ9 mutant cells and found a small but significant increase in Aβ associated with the EVs; however, Aβ levels in the latter EVs were considerably lower than EVs released from PS1Δ9 mutant cells. Our findings therefore suggest that while EVs have some capacity to interact with extracellular Aβ, much of the Aβ associated with EVs released from neural cells is present prior to their release. Accumulating evidence suggests that endosomes are a major site of APP processing to generate Aβ species.^59^ Endosomes may therefore be relatively enriched in Aβ that may associate with the intraluminal vesicles that can then be released from cells as EVs (exosomes). It has been shown that aggregation of proteins on the plasma membrane can trigger membrane budding and release of EV containing high amounts of the aggregated protein.^60^ Tetraspanins^61,62^ and syndecan heparan sulfate proteoglycans, together with Alix and syntenin,^63^ are involved in the formation and release of such EVs. Hence, it is possible that Aβ aggregation on the plasma and/or endosomal membranes results in Aβ secretion with EVs, and that such EVs are enriched in Aβ42 and Aβ40 in amounts and a stoichiometry^45,46^ that render them highly pathogenic.

In AD patients, active lysosomal proteases are associated with plaques and degenerating neurons, which also display other characteristics of lysosomal dysfunction including increased expression of hydrolases such as cathepsin D and accumulation of lysosomes.^64,65^ Familial AD PS1 mutations disrupt lysosomal acidification and autophagy.^66^ Cells in brain tissue samples from sporadic, late-onset AD patients also display a marked increase in immature autophagic vacuoles,^67^ a phenotype possibly related to lysosome impairment.^28^ Disruption of autophagy through heterozygous deletion of Beclin1 in an AD mouse model resulted in both intracellular accumulation of Aβ and increased amyloid plaque deposition,^68^ suggesting that autophagy is a major facilitator of Aβ clearance. However, crossing an AD mouse model with a mouse line defective in autophagy (ATG7 knockout mice) resulted in accumulation of intracellular Aβ and reduced Aβ secretion,^69^ suggesting that autophagy may also have a role in Aβ secretion. Previous studies have shown that mutations in PS1 cause lysosome impairment,^66^ EVs contain lysosomal proteins,^70^ and EV marker proteins such as Alix and flotillin-1 are present in AD amyloid plaques.^14,20^ We found that inhibition of autophagy (by exposure to bafilomycin A or CRISPR-Cas9-mediated knockdown of cathepsin D) in cells expressing mutant PS1 resulted in increased release of EVs with elevated amounts of Aβ42, suggesting that impaired autophagy may result in the release of pathogenic EVs in AD. These Aβ42-laden EVs are neurotoxic as indicated by their adverse effects on neuronal Ca^2+^ homeostasis, mitochondrial function, and vulnerability to excitotoxicity and apoptosis. Data from previous studies of postmortem brain tissue from sporadic, late-onset AD patients and of experimental cell culture and animal models, strongly support roles for impaired neuronal Ca^2+^ handling, mitochondrial dysfunction and apoptosis-like cell death in AD.^1,4,71^ Future studies involving animal models and human subjects will be required to elucidate the roles of Aβ-laden EVs in AD pathogenesis and their potential as a therapeutic target.

## Materials and methods

### Animals

APP/PS1 double-mutant transgenic mice (2xTgAD mice; B6C3-Tg(APPswe,PS1Δ9)85Dbo)^42^ and 3xTgAD mice (B6 129-Psentm1Mpm Tg, APPSwe, tauP301L1Lfa) mice^43^ were maintained under a 12 h light/12 h dark cycle with food and water available *ad libitum*. Mice were killed with an overdose of isoflurane anesthesia and blood was collected directly from the heart. All procedures were approved by the National Institute on Aging Animal Care and Use Committee and complied with NIH guidelines.

### Cell culture, experiments treatments, transfection and cell viability assays

Dissociated embryonic rat cortical cells cultures were established and maintained using methods similar to those described previously.^72^ Neurons were grown in polyethylenmine-coated plastic culture dishes or coverslips. Cultures were maintained at 37 °C (5% CO_2_/95% air atmosphere) in Neurobasal medium (Gibco 21103-049) containing B-27 supplement (Invitrogen) plus 1% antibiotic (Gibco 15240-062, Invitrogen). One quarter of the medium was replaced every 3 days and the cells were maintained for 7–10 days in culture prior to performing experiments. Human neuroglioma (H4TR) cells stably expressing wild-type (WT) PS1 or mutant PS1 (delta E9 mutation) under the control of a tetracycline responsive element were cultured in Dulbecco's modified Eagle medium (Gibco #12800-058) supplemented with 10% fetal bovine serum (Tet-tested, Thermo Fisher Scientific #SH30070.03T, Carlsbad, CA, USA), 50 μg/ml Zeocin (Invitrogen, #R250-01) and 2.5 μg/ml blasticidin (Invitrogen, #R210-01), and were maintained at 37 °C (5% CO_2_/95% air atmosphere). To induce expression of the mutant form of PS1, the cells were induced with 100 ng/ml tetracycline (Sigma-Aldrich #T7660, St Louis, MO, USA) for 3–5 days. Human neurons were differentiated from induced pluripotent stem cells that had been derived from fibroblasts taken from a patient with early-onset AD caused by a PS1 mutation (A246E) using methods described previously.^33^

Cells were grown to 50–60% confluency prior to transfection. The following plasmids were transfected using Lipofectamine LTX (Invitrogen 94756) according to the manufacturer’s protocol: Rab11-FIP3 CRISPR/Cas9 Double Nickase (sc-405735-NIC) and cathepsin D CRISPR/Cas9 (sc-400207). Bafilomycin A (Baf A) was dissolved in dimethylsulfoxide to generate a stock solution of 400 mmol/l and stored at −20 °C. When thawed, the Baf A was diluted 1:2,000 in cell culture medium. In order to examine the effects on cell viability, rat primary neurons were plated in a 96-well plate (30,000 cells per well) and treated as indicated. Neurons were exposed to EVs isolated from CSF or plasma (at a concentration calculated to result in a EV/neuron ratio of 100:1) or H4-derived EVs (EV/neuron ration of 300:1) for 48 h, and in some experiments 100 μmol/l glutamate was added after 24 h of EV treatment. Following 48 h EV incubation, 50 μl of medium was taken from each well and centrifuged at 300*g* for 5 min prior to measurement of LDH activity in the supernatant according to the manufacturer’s instructions (Roche 11644793001, Indianapolis, IN, USA). The MTT solution (Promega #G3581, Madison, WI, USA) was diluted 1 to 5, and 50 μl was added to each well. The MTT signal (light absorbance at 570 nm) was measured 0.5 to 4 h later using a Biotek Synergy H1 plate reader.

### Human CSF and plasma samples

CSF from six patients with sporadic, late-onset AD, MCI and age-matched neurologically normal subjects were obtained from the University of Kentucky AD Center. The latter CSF samples were drawn from living AD and MCI patients, and age-matched control subjects using standard National Alzheimer Coordinating Center and Alzheimer Disease Neuroimaging Initiative protocols.^73,74^ Samples were from: three female and three male AD patients (age range from 74–84 years); three female and three male MCI patients (age range from 65–92 years); and four female and two male control subjects (age range from 74–83 years). CSF from four additional AD and two MCI patients as well as plasma samples were obtained from the Clinical Research Unit of the National Institute on Aging (CRU-NIA) of Harbor Hospital (Baltimore, MD, USA). The diagnosis of AD used the criteria of probable AD as defined by the current NIA-AA criteria,^76^ although the diagnosis of MCI was based on the criteria set forth by the 2nd International Working Group on MCI^75^.

### Calcium imaging

Neurons that had been growing on glass coverslips for 8 days were treated with EVs at indicated concentrations for 2 days. Neurons were then incubated for 20 min in the presence of Fluo 8 (0.05 ng/ml) and then washed twice with Locke’s buffer. Neuronal Fluo 8 fluorescence was imaged using a Zeiss (Oberkochen, Germany) LSM 510 confocal microscope. The baseline fluorescence signal was recorded for 40–100 s, 100 μmol/l glutamate was then added, and the fluorescence signal was measured for at least an additional 400 s.

### Isolation of extracellular vesicles

CSF or plasma (0.5 ml) samples were centrifuged at 2,300*g* for 30 min to remove cell debris. The supernatant was transferred to a Beckman (Brea, CA, USA) ultracentrifugation tube (#326823) and diluted with 3 ml sterile filtered (0.22 μm filter Millex, EMD Millipore (Billerica, MA, USA) #SLG5033SS) phosphate-buffered saline (PBS) prior to centrifugation for 2.5 h at 120,000*g* (SW55 rotor *K*=48). The supernatant was carefully removed and the pellet was resuspended in lysis buffer for protein quantification or Neurobasal medium for functional assays. Rat and human neurons were grown without serum, and H4 neuroblastoma cells were washed with PBS and medium replaced with EV-depleted FBS prior to treatments and medium collection for isolation of EVs.^76^ EVs were isolated from approximately 30 ml of medium (~25–30 million cells). The medium was centrifuged at 500*g* for 10 min to remove dead cells, then the supernatant was centrifuged at 2,300*g* for 10 min to remove cell debris and the supernatant was stored at −20 °C. Once thawed (at room temperature), the medium was centrifuged at 120,000*g* for 2.5 h (SW28 rotor *K*=246). Following this the supernatant was removed for use as an EV-depleted control and the pellet was suspended in 3.5 ml sterile filtered PBS and centrifuged again at 120,000*g* for 2.5 h (SW55 rotor *K*=48).^29^ The supernatant was carefully removed and the pellet containing EVs was resuspended in either lysis buffer for protein quantification or Neurobasal medium for functional assays.

### EV labeling with PKH26/67

Membranes of EVs were labeled with the fluorescent probe PKH26 using a kit purchased from Sigma (#PKH26GL). EV pellets were suspended in 100 μl of buffer C prior to addition of 100 μl 2× PKH26 solution and incubated for 2 min, followed by addition of PBS containing 2% bovine serum albumin (Sigma #A-3912). The labeled EVs were centrifuged twice at 120,000*g* for 1 h (SW55i rotor K=48) with an intervening wash to ensure removal of unbound dye. The indicated amounts of EVs were added to the cells and incubated for the 4, 6 or 24 h. As a control, PBS with the same concentration of PKH26 was centrifuged under the same conditions and added to the cells as negative control. The internalization was quantified by confocal microscopy or using a fluorescence plate reader.

### Transmission electron microscopy

A drop of EV preparations (suspended in PBS) was added to a freshly ionized 300 mesh Formvar/Carbon-coated grid and incubated for 5 min to allow adherence of the EVs to the grid. The grid was then washed through 5–7 puddles of ddH_2_O; and negatively stained in 2% aqueous uranyl acetate for 30 s. Images were acquired using a FEI Tecnai G2 Spirit transmission electron microscope with TWIN Lens operating at 100 kV and an Olympus Soft Imaging System Megaview III digital CCD.

### Quantification of EV numbers

EV suspensions were diluted 1:20 or 1:200 to permit counting in the range of 3–15×10^8^ per ml with an NS500 nanoparticle tracking analysis system (NanoSight, Amesbury, UK). The EVs were visualized by their scattering of a focused laser beam and the collection of the scattered light using a standard optical microscope fitted with a CCD video camera. Five exposures of 20 s each were recorded from fields chosen randomly by a computer operating with NanoSight software, Malvern Instruments (Malvern, United Kingdom), which enables measurement of EV size and numbers.

### Immunofluorescence, microscopy and image analysis

Cells were grown on glass coverslips in a 24-well plate. Following the indicated treatment, cells were washed twice with PBS and were fixed in a solution of 4% paraformaldehyde in PBS for 20 min. Fixed cells were incubated in blocking solution (0.3% Triton X-100 and 10% normal goat serum in PBS) for 30 min, and then incubated overnight at 4 °C with antibodies against cleaved-caspase 3 (D175, Cell Signaling, Danvers, MA, USA), MAP2 (Hm2, Sigma M9942), Aβ (6E10 SIG39320-200). The cells were then washed three times with PBS and incubated with fluorescently tagged anti-rabbit or anti-mouse secondary antibodies (Invitrogen) in blocking solution for 1 h at room temperature. The cells were then washed twice with PBS and, if indicated, were stained with 0.02% Thioflavin S (Sigma T-1892) for 8 min and then washed three times with 80% ethanol, twice with water and two times with PBS. Nuclei were stained with 4′,6-diamidino-2-phenylindole dihydrochloride (Sigma #32670) in PBS for 10 min. All coverslips were then washed with PBS and mounted on microscope slides in an anti-fade medium (Vector Laboratories, Burlingame, CA, USA). Images were acquired using a Zeiss LSM 510 confocal microscope with a ×40 objective. Quantification of the staining intensity and area, and evaluation of co-localization were performed using Fiji software.^75^

### Protein extraction and immunoblots

Cells were washed twice with sterile PBS and scraped into a 15 ml tube prior to centrifugation at 700*g* for 10 min. The supernatant was then removed and the pellet was resuspended in 100–200 μl of lysis buffer (M-PER) with protease inhibitors (Complete Mini, Sigma) and incubated on ice for 30 min before centrifugation at 10,000*g* for 25 min. The supernatant was stored at −80 °C. Protein extracts from EVs was prepared by resuspension of the EV pellet in lysis buffer (M-PER) with protease inhibitors, followed by vortexing for 10 s (samples were stored at −80 °C). After thawing on ice the samples were sonicated in a water bath for 5 min and centrifuged for 25 min at 10,000*g*. The supernatant was transferred to a new tube and used directly or stored at −80 °C. Equal amounts of protein for each sample were resuspended in sample buffer (Life NP0008) and analyzed by polyacrylamide gel electrophoresis (Life technologies NOVEX NP0302BOX or NP0321BOX) and subsequent immunoblotting. Immunocomplexes were detected by enhanced chemiluminescence (Pierce 32106). The following antibodies were used for immunoblots: monoclonal anti-Alix (3A9, Cell Signaling 0512015) ), polyclonal anti-FLOT1 (Abcam ab133497, Cambridge, UK), polyclonal anti-CD9 (H-110, Santa Cruz SC9148, Dallas, TX, USA), anti-Aβ (6E10 SIG39320-200), and anti-mouse or anti-rabbit secondary antibodies (Jackson Laboratories 715-036-151, 711-036-152, Bar Harbor, MA, USA).

### Statistical analysis

Results are expressed as mean and s.e.m. of the indicated number of EVs preparation or independent neuronal cultures. The differences in numbers of cells that responded to glutamate treatment was determined using *χ* ^2^-test. Comparisons of Aβ concentration and ratios between EVs and EV-depleted fluids was calculated by Mann–Whitney *U*-test, unless there were more than two groups and then one-way analysis of variance was used. Neurotoxicity, calcium imaging and Seahorse data were evaluated using two-way analysis of variance followed by the Bonferroni *post hoc* test. The correlation between the EVs Aβ concentration and their toxicity was determined by linear regression. These analyses were performed using the Prism software package (Graphpad Software, San Diego, CA, USA).
