# Supplementary material for: Extracellular vesicle-associated Aβ mediates trans-neuronal bioenergetic and Ca2+-handling deficits in Alzheimer’s disease models
Source: NPJ Aging Mech Dis. 2016 Sep 22;2:16019–. doi: 10.1038/npjamd.2016.19 (PMC5137253; doi:10.1038/npjamd.2016.19)
Supplement: Supplementary Figures [file npjamd201619-s1.pdf]

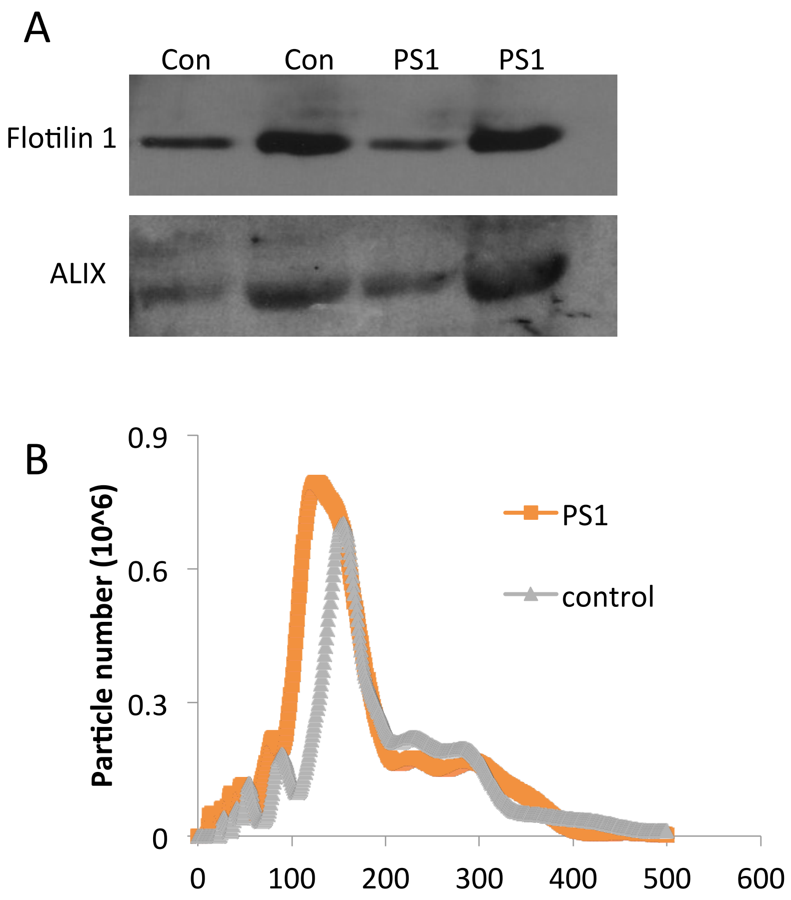

Supplemental Figure 1: **A.** Immunoblot analysis of EVs isolated from the medium of iPSC-derived neurons from two control subjects (Con) and two patients with PS1 mutations (PS1). The blots were probed with antibodies against two EV proteins, flotilin 1 and ALIX. **B.** EV size distribution as quantified by Nanosight tracking analysis.

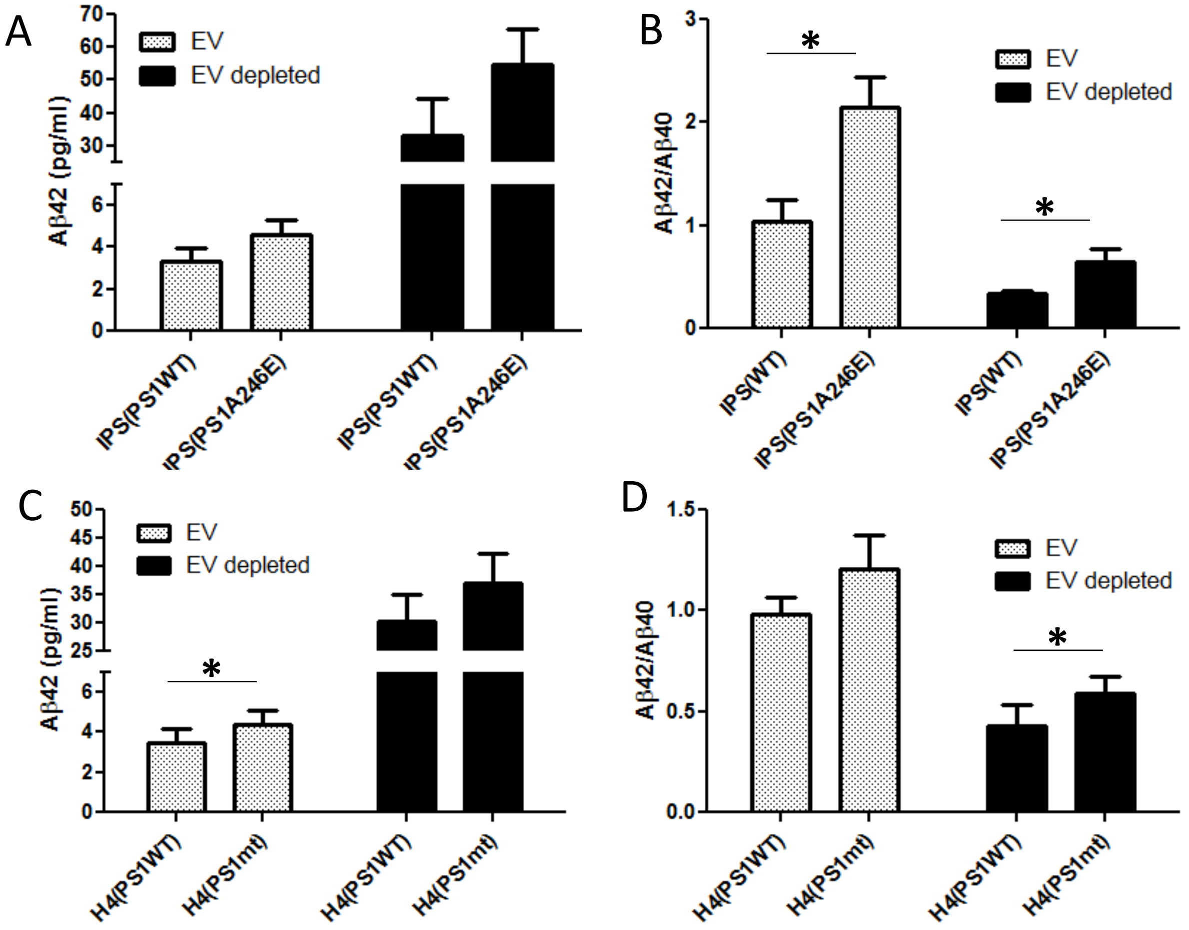

Supplemental Figure 2. The levels of Aβ42 and Aβ40 were significantly lower in EVs and EV-depleted culture medium samples of WT compared to AD models. **A and B**. MSD Aβ42 measurements (A) and Aβ42/40 ratio (B) for neurons differentiated from iPSCs generated from fibroblasts from either a control subject (WT) or a human PS1 mutant AD patient. **C and D**. MSD measurements of Aβ42 (C) and Aβ42/40 ratio (D) in EVs isolated from the medium of H4 cells expressing WT or mutant PS1. n = 4 WT and N=9 PS1 $*p<0.05$.

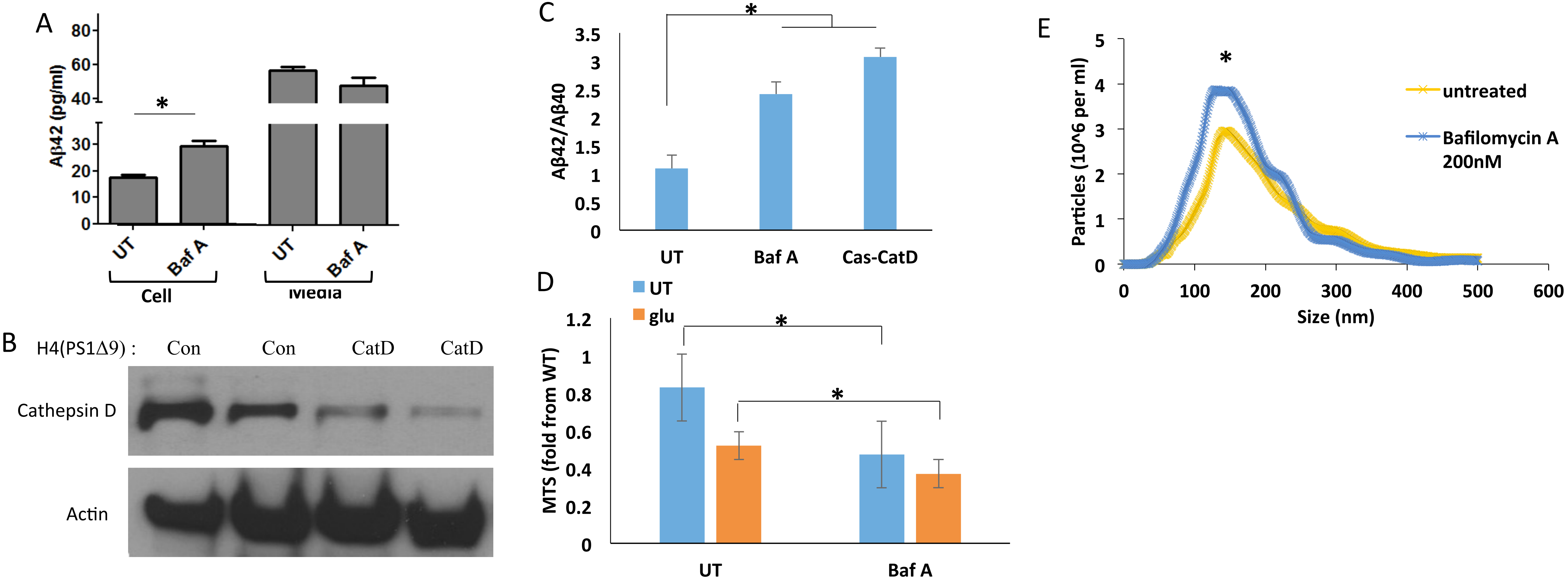

Supplemental Figure 3. Lysosome inhibition increases the EV-associated Aβ42/40 ratio in H4 cells expressing mutant PS1. **A.** Levels of Aβ42 in cell lysates and culture medium from cells that had been untreated or treated with bafilomycin A (Baf A, 200 nM for 24 hours. **B.** Immunoblot showing levels of cathepsin D in homogenates of H4 cells subjected to CRISPR Cas9-mediated knockdown of cathepsin D compared to control H4 cells. **C.** Aβ42/40 ratio in EVs released from H4 cells expressing mutant PS1. Cells that were either untreated (UT), or treated with bafilomycin A (Baf A; 200 nM) or cathepsin D-targeted CRISPR Cas 9 for 96 hours. **D.** Levels of MTS in rat primary neurons (7 days in culture) treated with EVs from H4PS1Δ9 cells that were treated with Baf A (200 nM) with or without co-treatment with 100 μM glutamate. N=4. **E.** Nanoparticle tracking analysis of EVs isolated from the medium of H4PS1Δ9 cells treated with bafilomycin A (200 nM) for 24 hours or untreated. N=3. *$p<0.05$

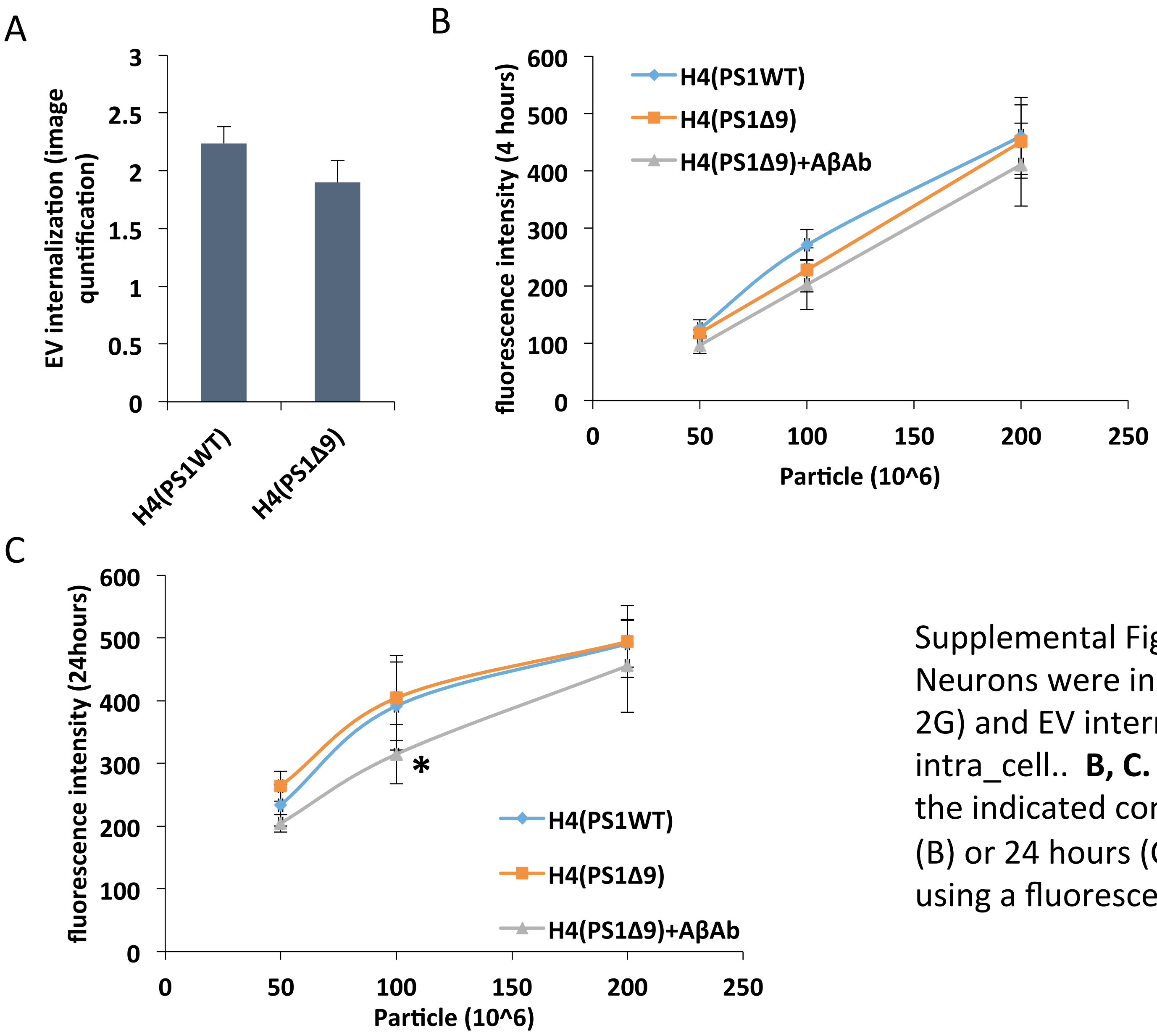

Supplemental Figure 4. EV internalization into rat primary neurons **A.** Neurons were incubated with PKH26-labeled EVs (see images in Figure 2G) and EV internalization was quantified using the image J plugin intra_cell.. **B, C.** Neurons were incubated with PKH26-labeled EVs at the indicated concentrations. The medium was removed after 4 hours (B) or 24 hours (C) and the internalized fluorescence was quantified using a fluorescence plate reader. N=3; *p<0.05 two way ANOVA

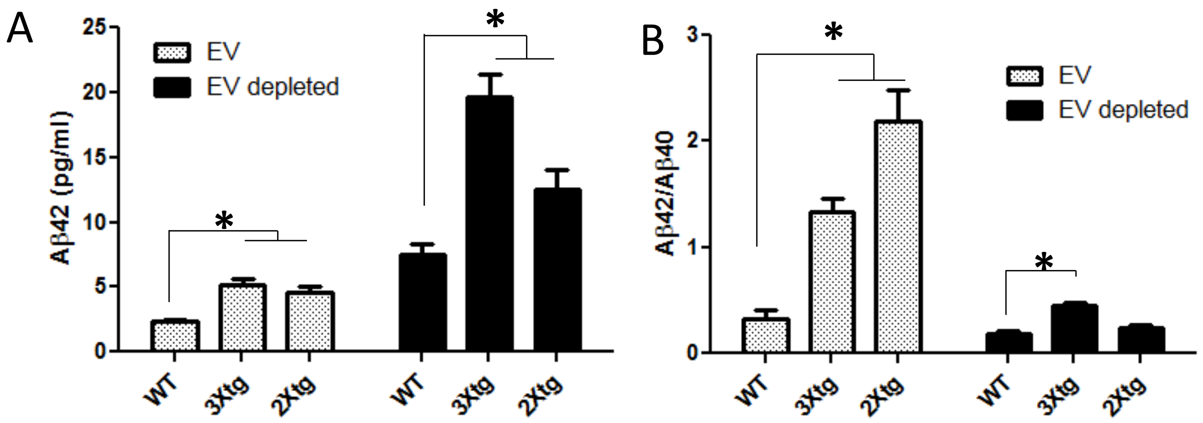

Supplemental Figure 5: **A and B**. Aβ42 (A) and the Aβ42/40 ratio (B) in plasma-derived EVs, and EV-depleted plasma from wild type mice (WT), 2xTg AD mice (2xTg) and 3xTg AD mice (3xTg). N= six 2xtg mice, five 3xtg mice, and 11 age-matched WT mice. *$p<0.05$.

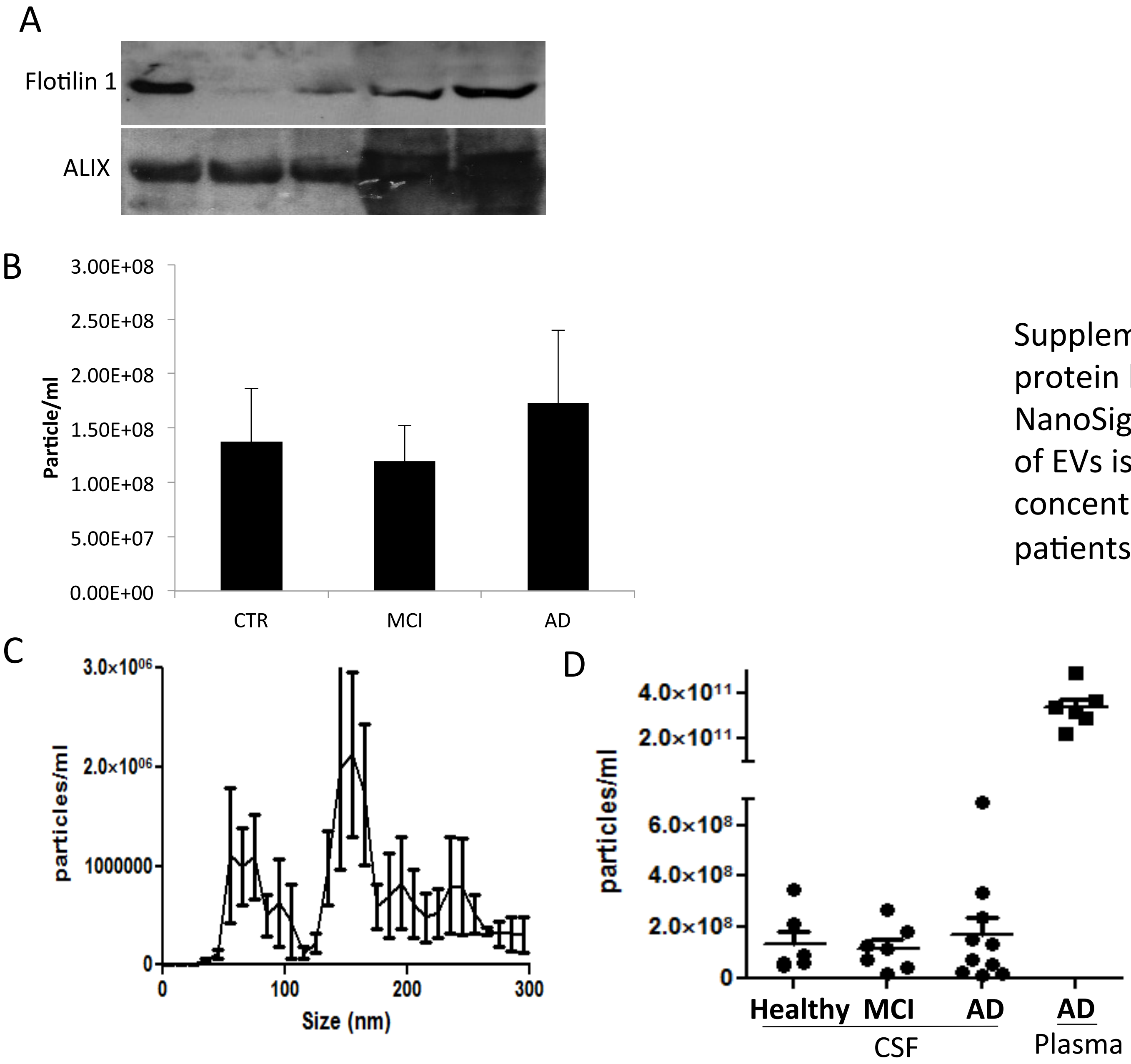

Supplemental Figure 6. **A.** Immunoblot analysis of Flotilin 1 and ALIX protein levels in EVs derived from CSF of 5 AD patients. **B and C**. NanoSight quantification of vesicle number (B) and size distribution (C) of EVs isolated from AD patient CSF. **D**. Scatterplot showing EV concentration in CSF samples from healthy control subjects, MCI patients and AD patients, and plasma of AD patients.

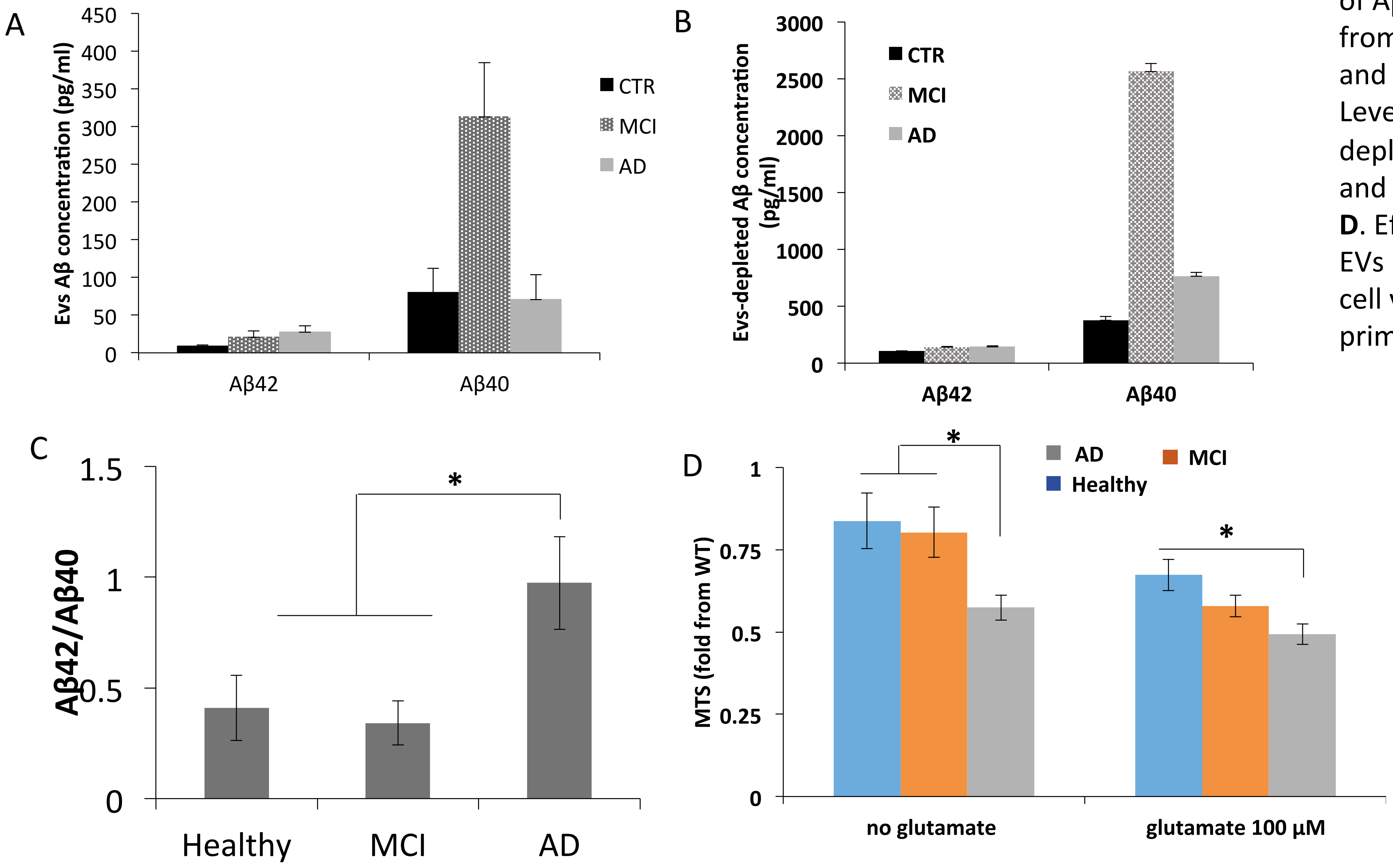

Supplemental Figure 7. **A**. Levels of Aβ42 and Aβ40 in EVs isolated from the CSF from control subjects and MCI and AD patients (n = 6). **B**. Levels of Aβ42 and Aβ40 in EV-depleted CSF from control subjects and MCI and AD patients (n = 6). **D**. Effects of patient CSF-derived EVs (100 particles per neuron) on cell viability (MTS assay) in rat primary neuronal cultures.

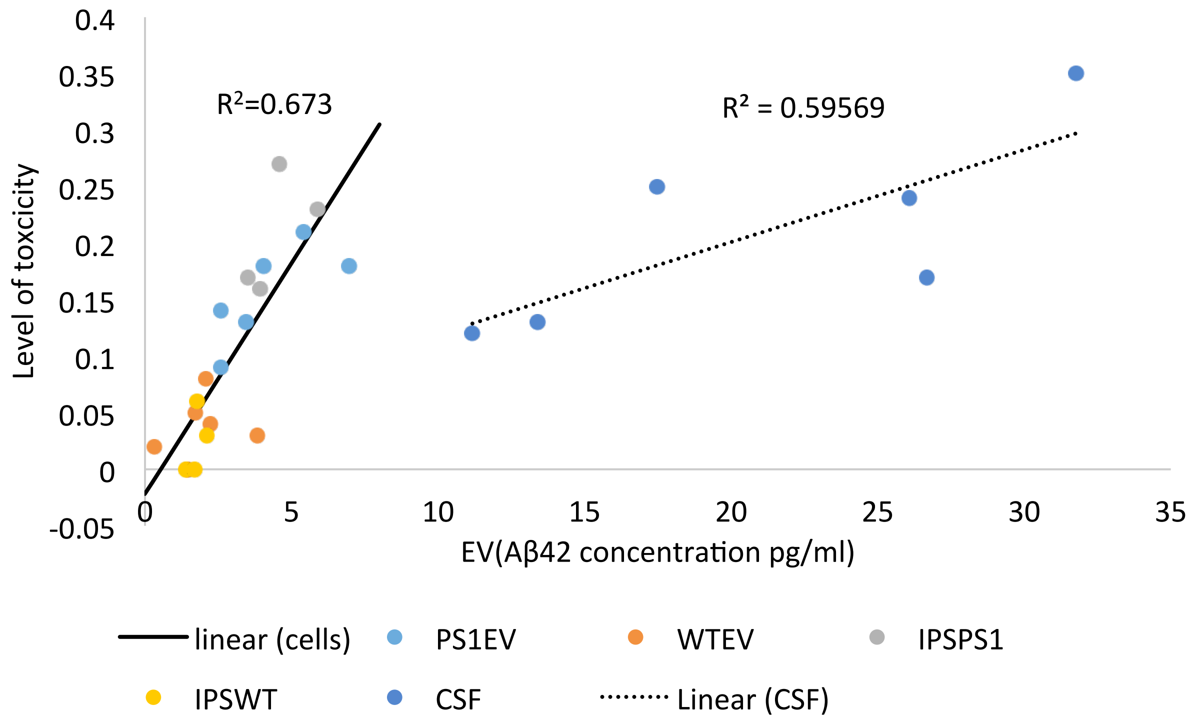

Supplemental Figure 8. EV-associated Aβ42 load correlates with EV-induced toxicity in cultured neurons across multiple AD models. EVs derived from H4 PS1d9 cells (PS1EV, light blue), H4 Psn1WT cells (WTEV, orange), iPSC-derived WT neurons (IPSWT, yellow), iPSC-derived PS1 AD neurons (IPSPS1, grey) and AD patient CSF (CSF, dark blue).
